# Costs of managing adverse events in the treatment of first-line metastatic renal cell carcinoma: bevacizumab in combination with interferon-*α*2a compared with sunitinib

**DOI:** 10.1038/sj.bjc.6605417

**Published:** 2009-11-17

**Authors:** G Mickisch, M Gore, B Escudier, G Procopio, S Walzer, M Nuijten

**Affiliations:** 1 Center of Operative Urology, Bremen 28277, Germany; 2 The Royal Marsden Hospital, London SW3 6JJ, UK; 3 Institut Gustave Roussy, Villejuif 94805, France; 4 Fondazione ‘IRCCS ‘Istituto Nazionale dei Tumouri', Medical Oncology, Milan 20133, Italy; 5 F. Hoffmann-La Roche Ltd, Basel CH-4070, Switzerland; 6 Erasmus University, Rotterdam 1546 LG, The Netherlands

**Keywords:** adverse events, bevacizumab, cost, management, sunitinib

## Abstract

**Background:**

Bevacizumab plus interferon-*α*2a (IFN) prolongs progression-free survival to >10 months, which is comparable with sunitinib as first-line treatment of metastatic renal cell carcinoma (RCC). The two regimens have different tolerability profiles; therefore, costs for managing adverse events may be an important factor in selecting therapy.

**Methods:**

Costs of managing adverse events affecting patients with metastatic RCC eligible for treatment with bevacizumab plus IFN or sunitinib were evaluated using a linear decision analytical model. Management costs were calculated from the published incidence of adverse events and health-care costs for treating adverse events in the United Kingdom, Germany, France and Italy.

**Results:**

Adverse event management costs were higher for sunitinib than for bevacizumab plus IFN. The average cost per patient for the management of grade 3–4 adverse events was markedly lower with bevacizumab plus IFN compared with sunitinib in the United Kingdom (€1475 *vs* €804), Germany (€1785 *vs* €1367), France (€2590 *vs* €1618) and Italy (€891 *vs* €402). The main cost drivers were lymphopaenia, neutropaenia, thrombocytopaenia, leucopaenia and fatigue/asthaenia for sunitinib; and proteinuria, fatigue/asthaenia, bleeding, anaemia and gastrointestinal perforation for bevacizumab plus IFN.

**Conclusion:**

The costs of managing adverse events are lower for bevacizumab plus IFN than for sunitinib. The potential for cost savings should be considered when selecting treatments for RCC.

Renal cell carcinoma (RCC) is the most common cancer of the kidney, with an incidence of 40 000 (3.1% of all cancer cases) annually and accounting for 26 000 deaths (2.3% of all cancer deaths) in Europe (Ferlay *et al*, 2007). The treatment landscape for metastatic RCC is changing, as a greater understanding of the biological processes involved in RCC development and growth, in particular the function of tumour angiogenesis, supports the development of more specific and effective therapies.

Bevacizumab (Avastin) is a humanised monoclonal antibody that precisely inhibits vascular endothelial growth factor (VEGF), the key mediator of tumour angiogenesis. In combination with cytotoxic chemotherapy, bevacizumab has demonstrated significant clinical benefits in several tumour types including metastatic colorectal (Hurwitz *et al*, 2004; Saltz *et al*, 2008), breast (Miller *et al*, 2007; Miles *et al*, 2008) and non-small-cell lung cancer (Sandler *et al*, 2006; Manegold *et al*, 2007). In metastatic RCC, initial phase II trials demonstrated that bevacizumab monotherapy is active and well tolerated in previously treated and treatment-naive patients (Yang *et al*, 2003; Bukowski *et al*, 2007). A large international, double-blind, randomised, controlled phase III trial (AVOREN) demonstrated that first-line bevacizumab combined with interferon-*α*2a (IFN, Roferon) significantly improved median progression-free survival (PFS: 10.2 *vs* 5.4 months; hazard ratio (HR)=0.63, *P*=0.0001) and objective response rate (ORR: 31 *vs* 13%, *P*=0.0001) compared with IFN plus placebo (Escudier *et al*, 2007). On the basis of these positive data, bevacizumab combined with IFN is approved in Europe for the first-line treatment of patients with advanced and/or metastatic RCC. A second phase III study, conducted in the United States (CALGB 90206), confirmed the significant benefit of bevacizumab and IFN *vs* IFN, with improvements in median PFS (8.5 *vs* 5.2 months; HR=0.71, *P*<0.001) and ORR (26 *vs* 13%, *P*<0.001) (Rini *et al*, 2008).

A number of other novel biological agents are available for the treatment of metastatic RCC, including sunitinib malate (Sutent), an orally active inhibitor of multiple receptor tyrosine kinases including VEGF receptors 1–3 and platelet-derived growth factor receptor-*α* and -*β* (Mendel *et al*, 2003). Sunitinib is also approved for first-line treatment of metastatic RCC on the basis of an open-label phase III trial showing significant improvement in ORR (47 *vs* 12%, *P*<0.0001) and median PFS (11.0 *vs* 5.0 months; HR=0.539, *P*<0.001) when compared with IFN (Motzer *et al*, 2007).

The results of these recent phase III trials in RCC suggest that the efficacy of bevacizumab plus IFN is comparable with that of sunitinib in the first-line treatment setting (Escudier *et al*, 2007; Motzer *et al*, 2007; Coppin *et al*, 2008). However, clinical data suggest that the two regimens have different tolerability profiles with respect to the type, severity and frequency of adverse events experienced by patients (Figure 1). These differences in the tolerability profiles of bevacizumab and sunitinib most likely reflect their different mechanisms of action.

The most frequently reported grade 3–4 adverse events in phase III trials of bevacizumab plus IFN include fatigue and asthaenia, hypertension, anorexia, bleeding, pyrexia and proteinuria; the majority of these are mild to moderate and manageable, and only a low incidence of grade 3–4 events is observed (Escudier *et al*, 2007; Rini *et al*, 2008). A retrospective subgroup analysis of the AVOREN trial has shown that the tolerability of the regimen is improved when lower doses of IFN are used in combination with bevacizumab (Melichar *et al*, 2008): IFN dose reduction led to a substantial decrease in the incidence of grade 3–4 adverse events 6 weeks after dose reduction compared with 6 weeks before dose reduction (18 *vs* 44%), while efficacy was maintained.

The most frequently reported grade 3–4 adverse events reported with sunitinib as first-line treatment of metastatic RCC include diarrhoea, vomiting, hypertension, hand-foot syndrome, leucopaenia, neutropaenia, thrombocytopaenia and mucositosis (Motzer *et al*, 2007; Negrier *et al*, 2008). The majority of adverse events associated with sunitinib are managed by sunitinib dose reduction or withdrawal (Sutent SmPC).

The development of severe adverse events is likely to require additional treatment and/or hospitalisation. Adverse event management costs, particularly hospitalisation, create an additional demand on health-care resources. Thus, when making a treatment choice for first-line RCC, the costs of managing adverse events are an important consideration from the perspective of health-care providers and physicians. Currently, there are few published data relating to management costs of adverse events in patients with metastatic RCC. This paper presents the results of a cost analysis to assess the estimated costs of managing adverse events associated with bevacizumab plus IFN compared with those associated with sunitinib for first-line treatment of RCC in the United Kingdom, Germany, France and Italy.

## Materials and methods

An Excel-based linear decision analytical model was developed to calculate and compare the costs of management of all grades of adverse events according to standard clinical practice for bevacizumab plus IFN and sunitinib used as first-line treatment of metastatic RCC. The model was populated using the total incidence of grade 1–4 adverse events reported in phase III trials in this disease setting (Escudier *et al*, 2007; Motzer *et al*, 2007), and with cost data associated with the management of adverse events from the perspective of health-care providers in the United Kingdom, Germany, France and Italy.

Cost data for the United Kingdom were obtained from a review of the published literature in the MEDLINE database using the adverse event description ‘type of adverse event’ and ‘cost’ and ‘UK’ and ‘RCC’. If this search strategy was unsuccessful, alternative search strategies were ‘type of adverse event’ and ‘cost’ and ‘UK’ and ‘oncology’ or ‘type of adverse event’ and ‘cost’ and ‘UK’. The same search terms were also used to obtain cost information from the UK National Institute for Health and Clinical Excellence (NICE). The primary perspective of this analysis was the same as that of the UK National Health Service.

Cost data for Germany were calculated from the diagnosis-related group (DRG) funding system catalogue (2008) and the from Einheitlicher Bewertungsma*β*stab (EBM) catalogue 2008 (Diagnosis Related Groups in Germany 2008; G-DRG system 2008; Einheitlicher Bewertungsma*β*stab für ärztliche Leistungen, Kassenärztliche Bundesvereinigung Berlin, 2008). Cost calculations in the DRG system are all inclusive, reflecting the costs of medicines, staff and maintenance, and provide an indication of the total cost for treating adverse events assuming that there is no severe underlying disease or complication. The EBM catalogue assigns points and costs per point for physician activities associated with costs of ambulatory treatment, but does not include medication costs. In this study, EBM points were assigned a value of €0.05; thus, 900 physician points corresponded to a cost of €4.50. To take into account the 2009 changes in the German EBM system, a point value of 3.5 cents was also applied.

In France, the cost of drugs was obtained from the Banque Claude Bernard database (Banque Claude Bernard, Group Cegedim, Boulogne Billancourt, France, 2007) and from the Pharmacie centrale des Hopitaux de Paris (APHP) (Pharmacie centrale des Hôpitaux de Paris, France, January, 2007). Costs for laboratory tests and examinations were derived from official tariff lists (Classification Commune des Actes Médicaux, CCAM V10, 2007; Table Nationale de codage de Biologie – Version 26 December, 2007). Hospitalisation costs (mean cost per stay) were estimated using the French DRG hospital database and the Etude Nationale de Couts (ENC) 2006 (Base de données PMSI, 2006; Etude Nationale de Coûts, 2006).

The assessment for Italy used information on the cost of treating adverse events from the report of a Delphi panel of experts from five clinical practices (Capri *et al*, 2007), from the Italian national DRG tariff (Conferenza delle Regioni e Province Autonome) and data from studies by Nuijten *et al* (2002) and Jansen *et al* (1997).

The base-case analysis conducted using the decision analytical model included all grades of adverse events, using as a threshold the cumulative total of events responsible for ⩾80% of total management costs. An additional scenario analysis was also conducted based only on the costs of managing grade 3–4 adverse events. As modelling studies are associated with uncertainty associated with input parameters, the robustness of the model was tested using sensitivity analyses based on varying hospitalisation costs within plus or minus 10% and by excluding the two most costly adverse events for bevacizumab plus IFN and with sunitinib. In Germany, medication costs are not included in the EBM cost estimates. Therefore, in Germany only, additional sensitivity analyses were conducted: first, 5% was added to the ambulatory medication costs to capture the costs of medications used to manage adverse events, and second, the effect of assigning a cost of €0.03 or €0.06 per EBM physician point was investigated.

## Results

### United Kingdom, Germany and France

**Figure 2:**
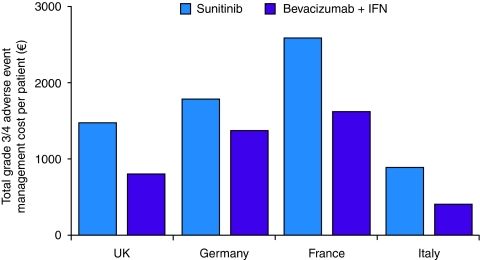
Total average cost per patient of managing grade 3–4 adverse events associated with bevacizumab plus IFN or with sunitinib in patients with metastatic RCC. IFN=interferon-*α*2a; RCC=renal call carcinoma. ^a^Motzer *et al*, 2007; ^b^Negrier *et al*, 2008; ^c^Escudier *et al*, 2007.

The average cost per patient of managing all-grade and grade 3–4 adverse events varied across the countries assessed (Table 1, Figure 2). The linear decision analytical model demonstrated that for all-grade and for grade 3–4 adverse events, management costs per patient were higher for sunitinib than for bevacizumab plus IFN in the United Kingdom, Germany and France (Mickisch *et al*, 2008). All-grade adverse event management costs per patient in the United Kingdom, Germany and France, respectively, were €1309, €1477 and €1957 for bevacizumab plus IFN and €2350, €2071 and €5127 for sunitinib. These differences represent potential cost savings of €1041 (44%), €594 (29%) and €3170 (62%) per patient in the United Kingdom, Germany and France, respectively, for patients with metastatic RCC treated with bevacizumab plus IFN compared with sunitinib.

A similar trend of higher management costs per patient with sunitinib compared with bevacizumab plus IFN was observed for grade 3–4 adverse events (Figure 2). In the United Kingdom, Germany and France, respectively, the management costs per patient for grade 3–4 adverse events were €804, €1367 and €1618 for bevacizumab plus IFN and €1475, €1785 and €2590 for sunitinib. These differences represent the opportunity for cost savings of €671 (45%), €418 (23%) and €972 (38%) per patient in the United Kingdom, Germany and France, respectively, for patients with metastatic RCC treated with bevacizumab plus IFN compared with sunitinib.

**Figure 3:**
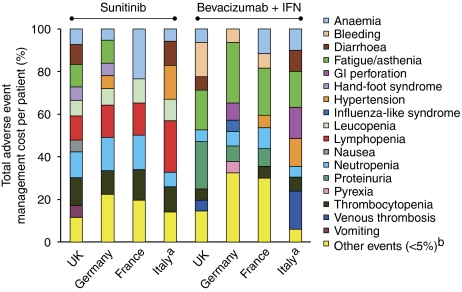
Cost distribution for management of adverse events with sunitinib and bevacizumab plus IFN in the United Kindgdom, Germany, France and Italy. ^a^On the basis of costs of managing grade 3–4 costs only; ^b^adverse events with proportional cost <5% are grouped. IFN=interferon-*α*2a; GI=gastrointestinal.

The main drivers of adverse event management costs for sunitinib and bevacizumab plus IFN were generally consistent across the countries examined (Figure 3). Neutropaenia, lymphopaenia, thrombocytopaenia, fatigue/asthaenia and anaemia were the main drivers of management costs associated with sunitinib. In contrast, proteinuria and fatigue/asthaenia were the main drivers of management costs associated with bevacizumab plus IFN, although bleeding, gastrointestinal (GI) perforation, anaemia and neutropaenia were shown to be associated with high costs in individual countries.

Sensitivity analyses based on a 10% difference in hospitalisation costs and excluding the costs of treating the principal adverse events associated with treatment were consistent with the overall analyses and demonstrated that bevacizumab plus IFN remained the least expensive treatment with respect to costs for managing adverse events compared with sunitinib (Table 2). Additional sensitivity analyses conducted in Germany showed that, even after taking into account medication costs, physician costs and a point value of 3.5 cents, bevacizumab plus IFN remained the least expensive treatment with respect to costs for managing adverse events compared with sunitinib. These sensitivity analysis findings demonstrate that the analytical model was robust.

### Italy

The results of the original analysis for the United Kingdom, Germany and France indicated that the vast majority of management costs for adverse events was associated with the development of grade 3–4 events. On this basis, only grade 3–4 adverse events were assessed for management costs in Italy (Procopio *et al*, 2008).

The average cost per patient of managing grade 3–4 adverse events for sunitinib (€891) was higher than that for bevacizumab plus IFN (€402); this difference represents an average cost saving of €489 (55%) per patient (Figure 2). Consistent with the results in the United Kingdom, Germany and France, sunitinib and bevacizumab plus IFN had different main drivers of adverse event management costs. Lymphopaenia, hypertension, thrombocytopaenia, diarrhoea and leucopaenia were the main drivers for sunitinib treatment. In contrast, venous thrombosis, fatigue/asthaenia, GI perforation and hypertension were the main drivers of adverse event management costs for bevacizumab.

Sensitivity analyses were consistent with the main results and confirmed the cost savings of bevacizumab plus IFN in Italy. A 10% difference in hospitalisation costs and excluding the costs of treating the principal adverse events associated with sunitinib and bevacizumab plus IFN showed cost savings that were consistent with those of other countries (Table 2).

## Discussion

The treatment of metastatic RCC has been transformed by the recent introduction of molecularly targeted agents. Sunitinib and bevacizumab, in combination with IFN, are approved for first-line treatment of patients with metastatic RCC and recent clinical studies demonstrate that they provide comparable levels of efficacy (Escudier *et al*, 2007, 2009; Motzer *et al*, 2007; Coppin *et al*, 2008). In an era in which there is a choice of effective therapy, a wide range of factors should be considered when selecting treatment, including overall tolerability profiles, ease of managing adverse events and costs associated with adverse event management.

Sunitinib and bevacizumab plus IFN have tolerability profiles that show important differences and are defined to varying degrees. Common adverse events associated with bevacizumab include hypertension, proteinuria and bleeding, whereas neutropaenia, leucopaenia, anaemia and hypertension are commonly associated with sunitinib. These differences and the relative definition of the drugs' tolerability profiles are presumably related to the mechanism of action of each of the agents. The common adverse events associated with bevacizumab seem to be related directly to its precise inhibition of VEGF signalling, with a defined mechanism of action described for most. In contrast, sunitinib is a multitargeted kinase inhibitor that has been shown to have activity against a range of cell signalling pathways, as well as against VEGF, and is therefore associated with both VEGF-specific and non-VEGF-specific toxicity. However, the pathophysiology of many sunitinib-associated adverse events, including rash, stomatitis, cardiac effects and ‘chemotherapy-like’ events such as neutropaenia, anaemia and hand-foot syndrome, remains to be fully elucidated.

This study used safety data from the two pivotal studies of bevacizumab plus IFN and sunitinib, which also provided independently confirmed PFS data showing that the two regimens have comparable efficacy (Escudier *et al*, 2007, 2009; Motzer *et al*, 2007). In contrast, two recent indirect comparison meta-analyses suggested that suntinib provides a superior PFS benefit (Mills *et al*, 2009; Thompson Coon *et al*, 2009). Unlike these meta-analyses, this study excluded data from CALGB 90206 because this trial was open label, did not report independently confirmed data, used North American centres that have limited experience of IFN and patients seemed to have a comparatively poorer prognosis.

In this study, the costs of managing adverse events associated with these therapies in the United Kingdom, Germany, France and Italy were considerably higher for sunitinib than for bevacizumab plus IFN. Modification of basic clinical and economic assumptions (hospitalisation costs and the main cost-driving adverse events) showed that the model remained stable over the entire range of plausible values for a given parameter, and was therefore robust. The main cost drivers for sunitinib were lymphopaenia, neutropaenia, thrombocytopaenia, leucopaenia and fatigue/asthaenia. In contrast, the main cost drivers for the management of adverse events associated with bevacizumab plus IFN were fatigue/asthaenia, proteinuria, bleeding, anaemia and GI perforation. The majority of the increased cost associated with sunitinib was related to the management of haematological toxicities, which accounted for little or none of the cost of managing adverse events associated with bevacizumab plus IFN; chemotherapy-related haematological adverse events are associated with an economic burden due to costly hospitalisation/treatment costs and negatively affect patient quality of life (Elliott, 1996; Liou *et al*, 2007). This study also suggests that sunitinib involves higher costs for the management of adverse events that patients perceive as troublesome and affect their everyday activities and well-being. For example, hand-foot syndrome with sunitinib often manifests 3–4 weeks after treatment initiation (Hutson *et al*, 2008); it occurs predominantly on pressure points on the hands and feet, making walking and manual and sporting activities difficult. In addition, GI disorders (e.g., diarrhoea and mucosal inflammation) are common with sunitinib and can often be uncomfortable and embarrassing for the patient, interrupting daily activities (e.g., work), as well as interfering with nutrition in patients who may already be compromised in this regard.

The management strategies available for dealing with adverse events due to sunitinib and bevacizumab plus IFN may also be relevant when choosing first-line treatment for RCC. Reducing the dose of IFN used in combination with bevacizumab substantially improves the tolerability and management of IFN-related adverse events, enabling patients to remain on therapy while maintaining efficacy (Melichar *et al*, 2008). These promising data derived from a retrospective analysis of AVOREN need to be confirmed, most likely from the ongoing prospective phase II trial of bevacizumab plus 3 MIU IFN (BEVLiN). The ability to improve tolerability by using lower doses of IFN in combination with bevacizumab provides even greater cost savings compared with sunitinib in the first-line treatment of metastatic RCC (Mickisch *et al*, 2009). Sunitinib-related adverse events are frequently managed by a dose reduction from 50 mg oral daily to 37.5 or 25 mg (Sutent SmPC), suggesting that lower doses of sunitinib are better tolerated than the recommended dose, which would presumably also reduce adverse event management costs. However, recent evidence shows that PFS and ORR with sunitinib correlate with drug exposure (Mendel *et al*, 2003; Houk *et al*, 2007), suggesting that dose reduction below a certain threshold may reduce efficacy and thereby potentially affect patient outcomes.

This study considered the cost of adverse event management for bevacizumab plus IFN and sunitinib in the United Kingdom, Germany, France and Italy. The average adverse event management costs varied across these countries but showed an overall trend of consistently lower costs for bevacizumab plus IFN versus sunitinib. Management costs varied between the countries, but country-specific cost calculations/tariff lists provide a probable explanation. In addition, direct costs from a prospective study may be needed to confirm the cost savings observed in this study. The linear decision analytical model used in this study used health-care costs according to standard clinical practice from a variety of sources and relied on adverse event data from individual clinical trials that may not be fully comparable or reflective of adverse events in daily clinical practice. In addition, the linear decision analytical model did not permit a statistical analysis of the cost differences between sunitinib and bevacizumab plus IFN nor did it permit an analysis of the effect of adverse events on treatment efficacy; additional studies may be needed to confirm these data. The study also highlights that there is no standardisation of treatment methods or costs across different countries, meaning that the potential effect of adverse event management costs should be assessed on an individual country basis. The poorly defined pathophysiology and management strategies of many sunitinib-associated adverse events may not have been captured by this analysis, that is, the potential of having to try different management approaches to identify the most effective may represent increased ‘hidden’ costs. Moreover, the analysis used costs for the management of haematological adverse events and hand-foot syndrome based on historical chemotherapy-associated costs. This could have underestimated the costs of managing sunitinib-associated adverse events. However, the utilisation of chemotherapy-associated costs will remain the standard approach until specific data for targeted therapies are available. Finally, this study did not consider drug administration costs or initial drug acquisition costs because country-specific initiatives may result in significant cost differences, making standardisation across countries difficult.

In conclusion, this study demonstrated that the different tolerability profiles of bevacizumab plus IFN and of sunitinib result in markedly lower adverse event management costs for bevacizumab plus IFN. As these regimens have comparable efficacy in the first-line treatment of metastatic RCC, the predicted cost savings in relation to the management of bevacizumab plus IFN could provide benefits to physicians and payers and may be an important consideration when making therapeutic choices. These findings raise important points and potential resource benefits in the current cost-conscious oncology environment, in which there is a demand for novel agents that provide the greatest benefit.

## Figures and Tables

**Figure 1:**
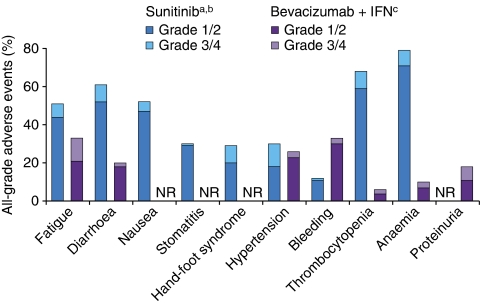
Frequency and severity of principal adverse events in patients with metastatic RCC treated with bevacizumab plus IFN or with sunitinib (Escudier *et al*, 2007; Motzer *et al*, 2007; Negrier *et al*, 2008). IFN=interferon-*α*2a; NR=not reported; RCC=renal call carcinoma.

**Table 1:** The cost (Euros) of grade 3–4 (grade 2) adverse event management per patient for bevacizumab plus IFN and sunitinib in patients with metastatic RCC in the United Kingdom, Germany, France and Italy

	**United Kingdom**			**Germany**			**France**			**Italy**		
		**Percent cost**			**Percent cost**			**Percent cost**			**Percent cost**	
**Adverse event**	**Cost per event** a	**Sunitinib**	**Bevacizumab + IFN**	**Cost per event** a	**Sunitinib**	**Bevacizumab + IFN**	**Cost per event** a	**Sunitinib**	**Bevacizumab + IFN**	**Cost per event** b	**Sunitinib**	**Bevacizumab + IFN**
Anaemia	2494 (112)	7.4	6.3	2194 (45)	5.3	4.6	1557 (3924)	23.4	11.6	1323	5.9	10.0
Anorexia	70 (70)	0	1.9	1860 (45)	0	4.5	36 (1911)	0	3.5	—	—	—
ATE	2494 (112)	0	1.7	2988^c^ (74)	0	1.7	523 (3592)	0	1.8	—	—	—
Bleeding	637 (637)	0	16.1	2376 (88)	0	6.2	79 (3592)	0	6.7	291	0	2.2
Chills	42 (42)	0.1	0	2946 (65)	1.5	0	78 (2236)	0.5	0	—	—	—
Decline eject fraction	1123 (1123)	1.3	0	2427 (65)	2.5	0	67 (4158)	1.7	0	1773	3.9	0
Depression	224 (224)	0	2.0	1860 (65)	0	4.1	147 (2072)	0	3.9	—	—	—
Diarrhoea	3207 (112)	9.1	6.4	1245 (45)	3.7	2.1	41 (2471)	2.8	2.9	1995	11.2	10.0
Dry skin	0 (112)	0	0	0 (45)	0.2	0	0	0	0	—	—	—
Dyspnoea	42 (42)	0	0.4	1269 (45)	0	0.8	78 (2236)	0	1.2	—	—	—
Epistaxis	1084 (112)	1.0	0	1209 (45)	0.8	0	32 (1328)	0.3	0	—	—	—
Fatigue/asthaenia	372 (372)	10.8	18.5	1860 (45)	10.7	28.6	36 (1911)	4.5	22.3	307	3.8	16.9
GI perforation	5929 (112)	0	4.5	16 929^c^ (0)	0	8.1	36 (8263)	0	4.2	5926	0	14.6
Hair colour changes	70 (70)	0.4	0	0 (45)	0.2	0	0	0	0	—	—	—
HFS	2589 (112)	6.2	0	2304 (45)	5.8	0	35 (881)	1.0	0	310	1.8	0
Headache	274 (274)	0.1	0.4	1527 (45)	0.9	2.5	32 (1217)	0.3	1.6	—	—	—
Heart failure	3293 (112)	0	0.8	5271^c^ (74)	0	0.9	67 (4158)	0	0.6	1773	0	1.2
Hypertension	21 (21)	0.2	0.4	1509 (45)	6.1	3.6	150 (2614)	4.5	5.8	1773	15.9	13.1
Influenza-like syndrome	42 (42)	0	0.8	3567 (45)	0	5.3	78 (2236)	0	4.3	—	—	—
Leucopaenia	1792 (112)	7.3	0	2376 (88)	7.9	0	498 (4432)	11.4	0	1802	10.1	0
Lymphopaenia	1792 (1792)	11.4	0	2376 (88)	15.2	0	498 (4432)	15.0	0	1802	24.2	0
Mucosal inflammation	495 (495)	4.2	0	1713 (45)	1.9	0	33 (33)	0.1	0	310	0.7	0
Myalgia	274 (274)	0.6	0	1623 (45)	0.8	0	0	0	0	—	—	—
Nausea	2803 (112)	5.5	0	1245 (45)	2.4	0	73 (602)	0.9	0	375	1.2	0
Neutropaenia	1792 (70)	12.0	5.7	2376 (88)	15.6	6.6	498 (4432)	16.2	9.8	511	6.8	5.0
Pain extremity	274 (274)	0.6	0	1623 (45)	0.9	0	35 (881)	0.2	0	—	—	—
Proteinuria	3929 (112)	0	22.0	1446 (87)	0	7.3	106 (2131)	0	8.2	137	0	2.5
Pyrexia	42 (42)	0.1	1.4	2946 (65)	1.6	5.3	78 (2236)	0.5	4.0	—	—	—
Rash	148 (148)	1.2	0	513 (45)	0.8	0	31 (1257)	0.6	0	310	0.7	0
Skin discolouration	70 (70)	0.5	0	0 (45)	0.2	0	31 (1257)	0.1	0	—	—	—
Stomatitis	495 (88)	1.1	0	1614 (65)	1.3	0	31 (2360)	0.6	0	—	—	—
Thrombocytopaenia	3372 (112)	13.2	5.4	2376 (0)	10.9	3.4	75 (3852)	14.4	5.5	1323	11.9	6.5
VTE	2246 (112)	0	5.1	2988^c^ (74)	0	3.5	74 (1305)	0	1.4	3591	0	17.9
Vomiting	2803 (112)	5.5	0	1245 (45)	2.7	0	73 (602)	0.8	0	375	1.7	0
Wound healing complications	148 (148)	0	0.1	3696 (77)	0	1.1	32 (2360)	0	0.7	—	—	—

Abbreviations: ATE=arterial thromboembolic events; GI=gastrointestinal; HFS=hand-foot syndrome; IFN=interferon-α2a; VTE=venous thromboembolic events.

a Grade 2 costs are shown in parentheses.

b Grade 3–4 costs only.

c Grade 3 cost lower than grade 4, grade 4 cost listed.

**Table 2:** Sensitivity analyses for the linear decision analytical model in the United Kingdom, Germany, France and Italy

	**Sunitinib costs**	**Bevacizumab + IFN costs**	**Cost savings (%)**
*United Kingdom*			
*Hospitalisation cost*			
Reduction 10%	€2202	€1230	€972 (44)
Increase 10%	€2497	€1391	€1106 (44)
			
*Exclude main sunitinib adverse event*			
Lymphopaenia	€2081	€1309	€772 (37)
Thrombocytopaenia	€2040	€1239	€801 (39)
Both adverse events	€1771	€1239	€532 (30)
			
*Exclude main bevacizumab* + *IFN adverse event*			
Proteinuria	€2305	€1023	€1282 (56)
Fatigue and asthaenia	€2097	€1067	€1030 (49)
Both adverse events	€2097	€780	€1317 (63)
			
*Germany*			
5% increase ambulatory treatment	€2085	€1482	€603 (29)
			
*Value of points*			
Three points	€2030	€1461	€569 (28)
Six points	€2275	€1555	€720 (32)
			
*Hospitalisation cost*			
Reduction 10%	€1893	€1340	€553 (29)
Increase 10%	€2250	€1613	€637 (28)
			
*Exclude main sunitinib adverse event*			
Neutropaenia	€1749	€1380	€369 (21)
Lymphopaenia	€1756	€1477	€2279 (16)
Both adverse events	€1434	€1380	€54 (4)
			
*Exclude main bevacizumab* + *IFN adverse event*			
Fatigue and asthaenia	€1848	€1054	€794 (43)
GI perforation	€2071	€1358	€713 (34)
Both adverse events	€1848	€935	€913 (49)
			
*France*			
*Hospitalisation cost*			
Reduction 10%	€4867	€1795	€3073 (63)
Increase 10%	€5386	€2118	€3267 (61)
			
*Exclude main sunitinib adverse event*			
Anaemia	€3926	€1730	€2196 (56)
Neutropaenia	€4296	€1764	€2531 (59)
Both adverse events	€3096	€1538	€1558 (50)
			
*Exclude main bevacizumab* + *IFN adverse event*			
Fatigue/asthaenia	€4896	€1521	€3375 (69)
Anaemia	€3926	€1730	€2196 (56)
Both adverse events	€3696	€1294	€2402 (65)
			
*Italy*^a^			
*Hospitalisation cost*			
Reduction 10%	€802	€362	€441 (55)
Increase 10%	€981	€442	€538 (55)
			
*Exclude main sunitinib adverse event*			
Lymphopaenia	€675	€402	€273 (40)
Hypertension	€750	€349	€401 (54)
Both adverse events	€533	€349	€184 (35)
			
*Exclude main bevacizumab* + *IFN adverse event*			
Venous thrombosis	€891	€330	€561 (63)
Fatigue/asthaenia	€858	€334	€524 (61)
Both adverse events	€858	€263	€595 (69)

Abbreviations: GI=gastrointestinal; IFN=interferon-α2a.

a On the basis of costs of managing grade 3–4 costs only.
